# Unusual death due to a bleeding from a varicose vein: a case report

**DOI:** 10.1186/1756-0500-5-488

**Published:** 2012-09-05

**Authors:** Kleio Fragkouli, Antigoni Mitselou, Vassiliki A Boumba, George Siozios, George T Vougiouklakis, Theodore Vougiouklakis

**Affiliations:** 1 Department of Forensic Medicine and Toxicology, University of Ioannina, Ioannina, Greece

**Keywords:** Varicose vein rupture, Massive hemorrhage, Autopsy, Medical emergency

## Abstract

**Background:**

Varicose veins are a common entity presenting a worldwide distribution. Although they are usually benign, sometimes are proved to be a threatening condition. Massive hemorrhage is an unusual complication of this common venous pathology that demands immediate medical intervention.

**Case presentation:**

We present a case of a 66-year-old woman found dead in her house surrounded by a large quantity of blood. Autopsy revealed a 7 mm ulcer on the internal surface of the left lower leg communicating with a varicose vein, signs of exsanguinations and liver cirrhosis. Toxicological analysis was negative.

**Conclusion:**

Massive hemorrhage from a ruptured varicosity is a severe medical emergency. Awareness of the risk of massive hemorrhage may provoke preventive treatment to be undertaken so as terminal loss of consciousness and a subsequent unattended death to be averted.

## Background

Varicose veins are a chronic clinical condition well known since antiquity and with a wide distribution [1]. According to a review of patients in Western countries, varicose veins affect one quarter to one third of all adults [2]. Although they are usually benign [3], severe varicosities may lead to major complications including edema, dermatitis, ulceration and severe bleeding [4]. The latter can be exacerbated when other factors - such as anticoagulant medications, alcohol, sedatives – contribute [5]. Cases where significant hemorrhage takes place may simulate arterial bleeding and represent medical emergencies and may lead to death if not treated immediately [6]. Those cases may mimic violent deaths because of the usually abundant amount of blood found at the scene of death [7].

We present an autopsy case of a 66-year-old woman who died due to fatal varicose vein rupture in the context of liver cirrhosis.

## Case presentation

**Figure 1:**
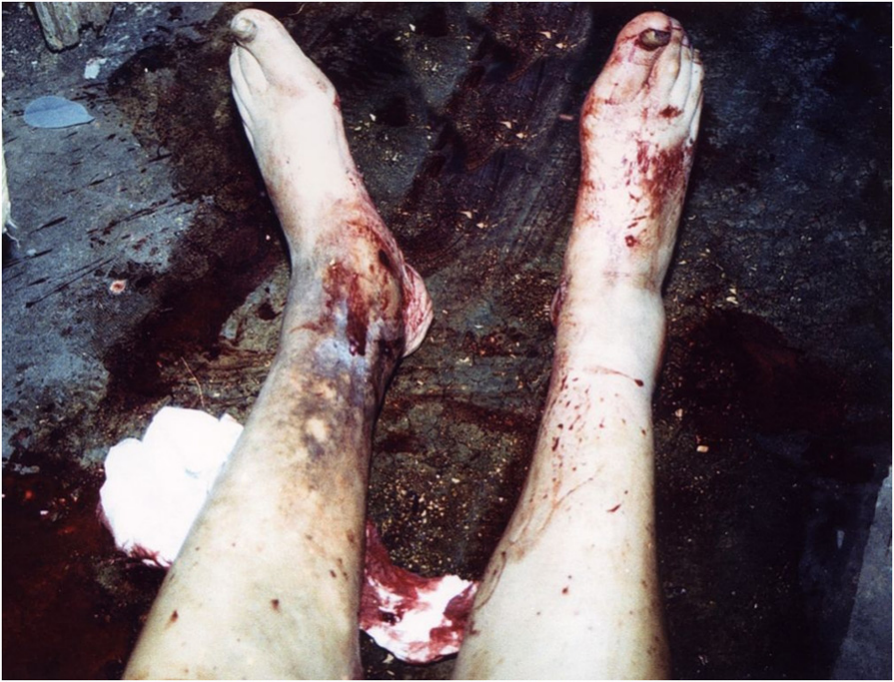
**The scene of death.** Victim’s low extremities surrounded by high quantity of blood and blood-soaked tissues.

A 66-year-old woman was found dead by a neighbor at her home, lying in a large quantity of blood. Multiple blood-soaked tissues were found around her body (Figure 1). The examination of the rest of the house revealed smears and smaller blood pools. The woman was living alone and her medical history was unknown.

**Figure 2:**
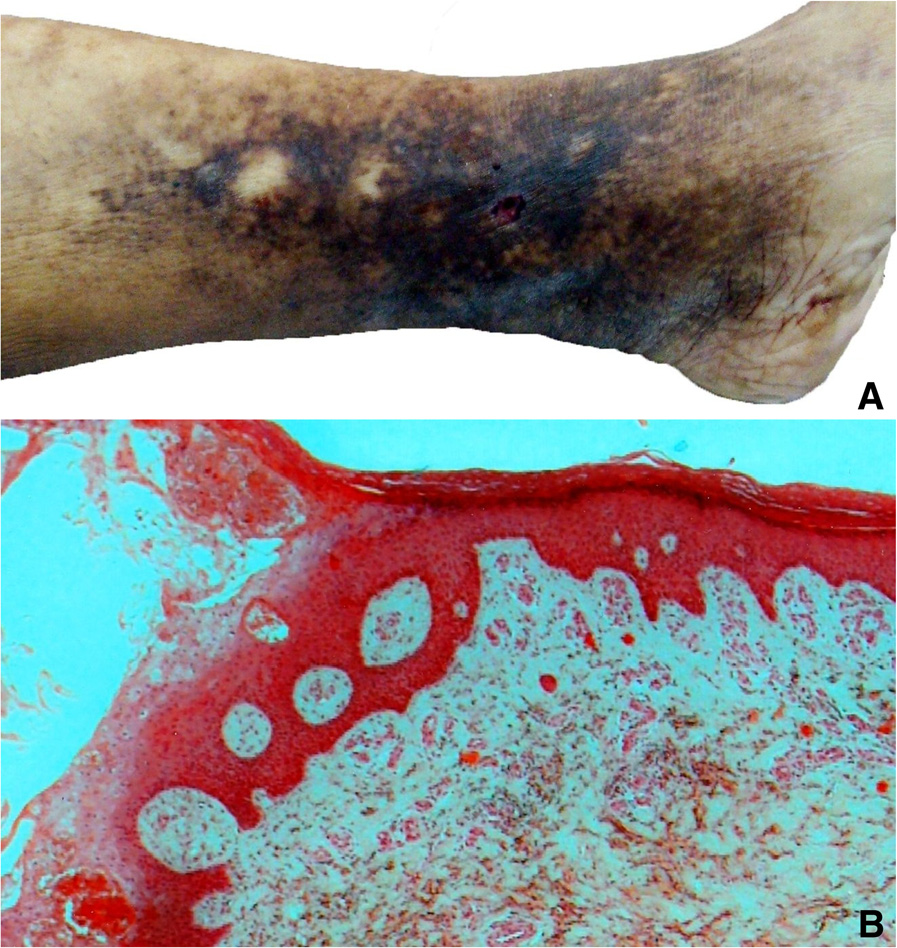
**The cutaneous ulcer and surrounded skin. (A)** Left lower leg showing skin pigmentation and induration and a 7 mm ulcer on internal surface, accompanied by an underlying varicosity perforation; **(B)** Microscopic image illustrating the ulcer margin covered by epidermis and fibrinous exudate and the underlying dermis with fibrous tissue, abundant macrophages and deposits of hemosiderin (Hematoxylin & Eosin x40).

During autopsy, sparely developed livers were observed on the body. The skin of her left lower leg showed significant edema, brownish-red discoloration and induration, compatible with chronic venous insufficiency. On the internal surface of left lower leg a 7 mm cutaneous ulcer was situated (Figure 2A). On dissection, the surrounding skin was remarkably firm, the ulcer margin was reddish and folded, whereas the base of the ulcer was red and with evidence of continuation with a superficial vein. No obvious traumatic lesions were observed.

Remarkable findings on internal examination included significant pallor of all the organs, subendocardial hemorrhages, a small yellowish, nodular and firm liver and an enlarged and congested spleen.

Histology revealed pulmonary edema and liver cirrhosis. Additionally, a defect in the epidermis with signs of continuity with a superficial vein was observed. The ulcer margin was covered by epidermis that abutted above, within or under the few fibrinous exudates. The underlying dermis contained numerous small and medium-sized blood vessels, filled with red blood and surrounded by thick organized fibrous cuffs. The remainder dermis presented fibrosis and abundant macrophages, as well as focally extravasated red blood cells and perivascular deposits of hemosiderin, a sign of repeated past dermal micro-hemorrhages (Figure 2B).

Toxicological analysis was negative.

Death was attributed to massive hemorrhage due to rupture of a varicose vein of the left lower leg.

## Discussion

Death from fatal hemorrhage due to varicose vein rupture is a well described phenomenon with a recognized representation in the literature [4-15]. Nevertheless, the incidence of this pathology as a cause of death presents a wide variety. Reported figures include a prevalence of 23 cases during a single year in England and Wales [8], and an incidence of <1:1000 in another autopsy series [4]. A total number of 66 cases have been described in reports since the phenomenon was described for the first time in 1973 [4-15]. The youngest person dying of fatal varicosity rupture up to date has been reported to be a 29-year-old man who was found deceased in his store lying in abundant blood [15].

Two types of ulceration related to fatal hemorrhage have been described: the acute perforative type, which is a small lesion (<5 mm) with almost no skin involvement; and the chronic ulceration, being a large lesion (10-100 mm) associated with skin pigmentation and induration and erosion into a superficial or deep vein of the leg [8]. In the present case, the ulcer was relatively small (7 mm) but there were also intense signs of longstanding venous insufficiency and stasis dermatitis, belonging finally in the chronic type. However, a combination with a minor trauma cannot be ruled out.

Characteristic features of deaths due to massive bleeding from a ruptured varicose vein have been mentioned. They include old age, social isolation, possible underlying medical conditions (e.g. restricted mobility, dementia), hemorrhage related to minor trauma, a rapid outcome and some associated features such as alcohol consumption or anticoagulant medications [4]. Another important risk factor is sclerotic changes of the vessel walls that may lead to spontaneous hemorrhage [13]. Additionally, predisposing factor to death has been reported to be a coexisting disease, such as ischemic heart disease [4]. Interestingly, in the case described herein, the presence of liver cirrhosis seems to have contributed to the fatal outcome. The chronic disturbance in the capacity of liver to produce the coagulant factors in the case of cirrhosis prevented the formation of a blood-clot to suppress the massive loss of blood, predisposing to significant hemorrhage and accelerating the death procedure.

In the cases of death due to varicosity rupture it is worth mentioning the victims’ failure to give themselves the adequate immediate medical care, e.g. direct pressure application and elevation of the bleeding limb. The victim in the present case tried to give herself a first aid, as bloodstained tissues were found around her at the place of death, but the coexisting liver pathology (cirrhosis) seems to have exaggerated hemorrhage, something that quickly resulted in unconsciousness and hastened death. Such a hemorrhage is definitely a medical emergency and requires prompt treatment. However, the possible lack of training and obtaining the right information relevant to this pathology, as well as the false appreciation of the importance of bleeding due to mental disease or alcohol ingestion, are the main factors that no first aids are given [4].

## Conclusion

Although varicose veins are a benign clinical pathology, they conceal the danger of rupture, especially if left untreated. The subsequent venous bleeding can be severely intensive and can quickly lead to a loss of consciousness and death. This case is remarkable from the forensic pathology point of view but also contains a preventive imperative. Awareness of the risk of massive hemorrhage may provoke preventive treatment to be undertaken. Therefore, therapists and general practitioners should bear in mind that whenever they treat a patient for varicose veins or any other pathology that might deteriorate varicosities, they should always consider the possibility of adverse interaction of the morbid entities leading to fatal bleeding of a varicose vein.

## Consent

Written informed consent was obtained from the patient daughter (next of kin since the patient is dead) for publication of this case report and any accompanying images. A copy of the written consent is available for review by the Editor-in-Chief of this journal.

## Competing interests

The authors declare that they have no competing interests.

## Authors’ contributions

KF wrote the manuscript, performed the literature search and contributed to autopsy. AM performed the histological examination and provided the micro-photograph. VB performed the toxicological analysis. GS and GTV contributed to autopsy and have been involved in drafting the manuscript. TV performed the autopsy and revised the manuscript. All authors read and approved the final manuscript.
